# Potassium Intake, Bioavailability, Hypertension, and Glucose Control

**DOI:** 10.3390/nu8070444

**Published:** 2016-07-22

**Authors:** Michael S. Stone, Lisa Martyn, Connie M. Weaver

**Affiliations:** 1 Department of Nutrition Science, College of Health and Human Sciences, Purdue University, West Lafayette, IN 47907, USA; stone59@purdue.edu (M.S.S.); lmartyn@tcd.ie (L.M.); 2 Human Nutrition and Dietetics, Dublin Institute of Technology, Dublin 2, Ireland

**Keywords:** potassium, bioavailability, hypertension, glucose control, diabetes

## Abstract

Potassium is an essential nutrient. It is the most abundant cation in intracellular fluid where it plays a key role in maintaining cell function. The gradient of potassium across the cell membrane determines cellular membrane potential, which is maintained in large part by the ubiquitous ion channel the sodium-potassium (Na+-K+) ATPase pump. Approximately 90% of potassium consumed (60–100 mEq) is lost in the urine, with the other 10% excreted in the stool, and a very small amount lost in sweat. Little is known about the bioavailability of potassium, especially from dietary sources. Less is understood on how bioavailability may affect health outcomes. Hypertension (HTN) is the leading cause of cardiovascular disease (CVD) and a major financial burden ($50.6 billion) to the US public health system, and has a significant impact on all-cause morbidity and mortality worldwide. The relationship between increased potassium supplementation and a decrease in HTN is relatively well understood, but the effect of increased potassium intake from dietary sources on blood pressure overall is less clear. In addition, treatment options for hypertensive individuals (e.g., thiazide diuretics) may further compound chronic disease risk via impairments in potassium utilization and glucose control. Understanding potassium bioavailability from various sources may help to reveal how specific compounds and tissues influence potassium movement, and further the understanding of its role in health.

## 1. Introduction

Potassium is an essential nutrient. It is the most abundant cation in intracellular fluid, where it plays a key role in maintaining cell function, particularly in excitable cells such as muscles and nerves. Because potassium is a major intracellular ion, it is widely distributed in foods once derived from living tissues. Potassium concentration is higher in fruits and vegetables than in cereals and meat. Salting foods and discarding the liquid induces sodium (Na+) for potassium (K+) exchange and reduces the potassium content of foods. Western dietary practices with higher consumption of cereal, low nutrient density processed foods and lower consumption of fruits and vegetables has led to a diet lower in potassium and higher in sodium in recent decades [1].

Recommended adequate intakes for potassium were set by the Food and Nutrition Board of the Institute of Medicine at 4700 mg/day [2]. This was largely based on meta-analyses of randomized, controlled trials investigating the effect of potassium supplementation on reducing blood pressure. Few Americans meet the recommended intakes; the average intake is 2591 ± 9 mg/day [3]. This large gap between potassium intakes and recommended intakes led to potassium being called a shortfall nutrient in the Dietary Guidelines for Americans [4].

Actual potassium requirements would vary with an individual’s genetics, blood pressure (BP) status, and sodium intake. Blood pressure is currently the primary criterion for determining potassium requirements, with blacks being more vulnerable to hypertension and more responsive to potassium supplementation than whites, hypertensive individuals more responsive to increasing potassium intakes than normotensive individuals, and potassium having a greater benefit for those consuming a high salt diet [1]. Other benefits of increasing potassium consumption may include improved glucose control, glucose intolerance and insulin resistance becoming a concern for hypertensive individuals prescribed to potassium wasting diuretics [5]. These differences support personalized nutrition approaches. Understanding movement of potassium within the body may help to improve these health outcomes.

Aside from host characteristics, recommended potassium intakes depend on bioavailability of potassium from foods. Generally, requirements for any nutrient are based on replacing losses from the body, adding in any demand for growth, and adjusting for absorption from the diet. However, recommended intakes for potassium were based on absorption from supplements because the first bioavailability study in any food, i.e., potato, was only recently reported [6]. The aims of this review are to discuss what is known about potassium bioavailability and metabolism and some of the consequences of deficiency.

## 2. Worldwide Potassium Intakes

### 2.1. United States of America

It is estimated that only 3% of adults and 10% of children under the age of five in the United States meet the adequate intake (AI) level for potassium [7,8]. However it should be noted that the US AI, set at 4700 mg/day for adults, is substantially higher than the World Health Organization’s (WHO) guidelines, which recommend 3150 mg/day for adults [9,10]. National Health and Nutrition Examination Survey (NHANES) data indicates that 99.2% of potassium in the US diet is naturally occurring, with the remaining 0.8% coming from fortified foods [3]. These naturally occurring sources include milk and other non-alcoholic beverages, as well as potatoes and fruit, which rank highest as sources of potassium intake among US adults [11].

### 2.2. Europe

Welch et al. [12] examined potassium intakes from 10 European countries and found that, for both men and women, Greece had the lowest average intakes at 3536 mg/day and 2730 mg/day respectively. The highest potassium intakes were found in Spain where the average intake for men was 4870 mg/day and for women was 3723 mg/day [12]. Meat and meat products and cereals and cereal products are the main contributors to potassium intake in Europe [12]. However, there is substantial geographical variation in the contribution of other potassium rich foods. It is estimated that fruit and vegetables provide 17.5% of total potassium intake in Nordic countries compared to 39% in Greece [12], while in the United Kingdom (UK), “vegetables and potatoes” are the largest contributors to potassium intake, providing 24.5% [13]. 

### 2.3. Asia

Despite an increase of 300 mg/day since 1991, potassium intakes in China remain poor at 1800 mg/day [14]. This does not meet the Chinese dietary reference intake (DRI) for potassium, which is set at 2000 mg/day, and is less than half of the World Health Organization (WHO) recommendation of 3150 mg/day [10,15]. Intakes are higher in Korea where the average potassium intake is 2900 mg/day yet still do not meet the WHO guidelines [16]. The primary potassium sources in the Korean diet are white rice, fruits and vegetables [16].

## 3. Internal and External Balance of Potassium

### 3.1. Potassium Tissue Movement

Total body potassium (K+) is estimated to be approximately 43 mEq/kg in adults, with only 2% of this found in the extracellular fluid. Most of the body potassium content is found in the intracellular space of skeletal muscle. Potassium is the primary intercellular cation and plays a key role in maintaining cell function, having marked influence on transmembrane electro-chemical gradients [17]. The gradient of potassium across the cell membrane determines cellular membrane potential, which, based on the normal ratio of intracellular to extracellular K+, is −90 mV. This potential difference is maintained in large part by the ubiquitous ion channel, the sodium-potassium (Na+-K+) ATPase pump. When activated, the Na+-K+ ATPase pump exchanges two extracellular K+ ions for three intracellular sodium (Na+) ions, influencing membrane potential based on physiological excitation or inhibition. These channels are partially responsible, along with the Na+-K+ chloride (Cl) symporter, and sodium-calcium (Ca) exchanger, for maintaining the potential difference across the resting cell membrane as well. Both resting membrane potential and the electro-chemical difference across the cell membrane are crucial for normal cell biology, especially in muscle and nervous tissue [17,18].

Distribution of potassium under normal physiological conditions is referred to as internal balance. Transmembrane electro-chemical gradients cause the diffusion of Na+ out of the cell and K+ into the cell. This process is reversed, and cellular potential difference is held constant, via the Na+-K+ ATPase pumps, mentioned previously. Na+-K+ ATPase pumps express B_2_ adrenergic receptors, which are highly stimulated by the circulating stress hormones catecholamines (epinephrine/norepinephrine) and insulin [17,18]. Subsequently, alterations in these hormone levels can effect cellular ion movement and serum potassium. Stimulation of both B_2_ adrenergic and insulin receptors leads to the cellular influx of potassium by increasing the activity of Na+-K+ ATPase pumps (primarily in skeletal muscle). Insulin binding to cell surface tyrosine kinase receptors (insulin substrate receptor-1; IRS1) also stimulates the translocation of intracellular glucose transport proteins (GLUT4 in muscle) facilitating the influx of glucose into the cell. Downstream activation of signaling cascades involving cyclic adenosine-mono-phosphate (cAMP), protein kinase A (Akt), and IRS1-phosphatidylinositide-3-kinase (PI3-K) dependent pathways facilitate both potassium and glucose uptake [18]. While hormones play an important role in the movement of potassium within the body, the concentration of other ions (inorganic and organic) are also influential in maintaining proper internal balance.

Metabolic acidosis caused by inorganic anions (mineral acidosis) can also stimulate potassium movement. The effect of acidemia on enhancing cellular K+ loss is not related to direct potassium–hydrogen (H+) ion exchange, but rather via action on transporters which normally regulate skeletal muscle pH [19]. The decrease in extracellular pH reduces the rate of sodium-H+ exchange and inhibits sodium (Na)–bicarbonate (HCO_3_) cotransport. The fall in intracellular Na+ reduces Na+-K+ ATPase activity, leading to decreased K+ influx and cellular K+ loses [17]. Additionally, a fall in extracellular HCO3 increases inward flux of chloride via upregulation of chloride (Cl)-HCO_3_ exchange, increasing K+-chloride cotransport and subsequent K+ efflux. In metabolic acidosis via organic anion accumulation, loss of potassium from the cell is much smaller. Accumulation here, through movement of both anions and H+ through monocarboxylate transporters (MCT; MCT1, MCT4), leads to a lower intracellular pH, stimulating the movement of sodium via Na+-H+ and Na+-HCO_3_ transporters. An increase of intracellular sodium maintains Na+-K+ ATPase activity, limiting the efflux of K+ [17]. Generally, metabolic acidosis (inorganic or organic) causes greater K+ efflux than respiratory acidosis, HCO3 being the primary anion accumulating in the cell to balance the influx of hydrogen ions [20]. Movement of cellular K+ varies similarly in response to different types of physiological alkalosis as well. In respiratory alkalosis, K+ influx is reduced compared to metabolic alkalosis, due to the efflux of cellular HCO_3_ [20]. Potassium supplementation is often given in a form with a higher acid load (e.g., potassium chloride), while dietary forms are more alkaline (potassium bicarbonate/citrate), possibly leading to a reduction in the overall potential renal acid load (PRAL) and reduced urinary calcium loss. [21].

### 3.2. Renal Potassium Handling

Approximately 90% of potassium consumed (60–100 mEq) is lost in the urine, with the other 10% excreted in the stool, and a very small amount lost in sweat [22]. Potassium has a higher ratio of dietary intake to extracellular pool size; only 2% of the total body K+ is distributed in ECF with the remaining distributed in the ICF of various tissues [23]. To meet the challenge of a high potassium meal the K+ homeostatic system is very efficient at clearing plasma K+ via an increase in renal K+ excretion. When dietary K+ intake increases or decreases the kidneys modulate excretion accordingly, ensuring the maintenance of plasma [K+]. In addition, with the administration of acute K+ loads, only approximately half of the dose appears in the urine after 4–6 h, suggesting that extrarenal tissues (e.g., muscle) play an important role in K+ homeostasis as well [23,24].

Potassium is freely filtered by the glomerulus of the kidney, with most of it being reabsorbed (70%–80%) in the proximal tubule and loop of Henle [22]. Under physiological homeostasis delivery of K+ to the nephron remains constant. Conversely, secretion of K+ by the distal nephron is variable and depends on intracellular K+ concentration, luminal K+ concentration, and cellular permeability [17,18]. Two major factors of K+ secretion/loss involve the renal handling of sodium and mineralocorticoid activity. Reabsorption in the proximal tubule is primarily passive and proportional to reabsorption of solute and water, accounting for ~60% of filtered K+ [25,26]. Within the descending limb of Henle’s loop, a small amount of K+ is secreted into the luminal fluid, while in the thick ascending limb, reabsorption occurs together with Na+ and Cl, both trans- and paracellularly. This leads to the K+ concentration of the fluid entering the distal convoluted tubule to be lower than plasma levels (~2 mEq/L) [26]. Similar to reabsorption in the proximal tubule, paracellular diffusion in Henle’s loop is mediated via solvent drag, while transcellular movement occurs primarily through the apical sodium-potassium-chloride (Na+-K-2Cl) co-transporter [17,26]. The renal outer medullary K+ channel (ROMK), also located on the apical membrane, mediates recycling of K+ from the cell to the lumen, sustaining the activation of the Na+-K-2Cl cotransporter and K+ reabsorption in the ascending limb [17,18]. The movement of K+ through ROMK induces a positive lumen voltage potential, increasing the driving force of paracellular K+ reabsorption as well. Na+-K+ ATPase pumps located basolaterally throughout the loop, maintain low levels of intracellular Na+ and further provide a favorable gradient for K reabsorption.

Major regulation of K+ excretion occurs in the late distal convoluted tubule (DCT) and the early connecting tubule [27]. In the early DCT luminal, Na+ influx is mediated by the apical sodium-chloride (Na+-Cl) co-transporter, and continues into the late DCT via the epithelial Na+ channel (ENaC). Both are expressed apically and are the primary means of Na+ reabsorption from the luminal fluid. Na+ reabsorption leads to an electro-chemical potential that is more negative than peritubular capillary fluid. This charge imbalance is matched by an increase in the aforementioned paracellular reabsorption of Cl from the lumen, as well as increases in Na+-K+ ATPase and ROMK activity [22]. Increased distal delivery of Na+ increases Na+ reabsorption, leading to a more negative luminal/plasma potential gradient and an increase in K+ secretion.

Aldosterone, the major mineralocorticoid in humans, increases K+ secretion by stimulating an increase in luminal Na+ reabsorption. Aldosterone directly increases the cellular concentration of Na+ via stimulation of ENaC expression and increased Na+-K ATPase and ROMK activity [22]. Aldosterone mediated increases in luminal Na+ uptake upregulate Na+-K+ ATPase activity indirectly as well. In addition the diurnal rhythm of aldosterone secretion may significantly influence its effect on renal K+ excretion, and should be taken into consideration with random (non-24 h) urine sampling [28]. Excessive extra-renal K+ losses are usually small, but can occur in individuals with diarrhea, severe burns, or excessive and prolonged sweating [18].

## 4. Potassium Bioavailability

Potassium is found in most plant and animal tissues, with fruits and vegetables having a higher nutrient density than cereals and animal foods. Potassium is intrinsically soluble and quickly dispersed in the luminal water of the upper digestive tract. The small intestine is the primary site of potassium absorption, with approximately 90% of dietary potassium being absorbed by passive diffusion [29]. Little is known about the bioavailability of potassium, with the majority of work being centered on the assessment of urinary potassium losses after potassium salt supplementation [30,31,32].

### Kinetic Modeling and Potassium Bioavailability

Many different models of potassium movement within the body have been proposed, each developed to fit various areas of biological interest. The complexity of each model varies, from early recommendations by the International Commission on Radiological Protection for evaluation of radiopotassium exposure limiting the body to one large mixed pool of potassium, to more complex anatomically related compartmentalization [33,34,35]. In one of the earliest schemes, Ginsburg and Wilde constructed a five compartment model, mathematically derived from murine data looking at tissue groupings (muscle/testes, brain/RBC, bone, lung/kidney/intestine, liver/skin/spleen) and their potassium exchange between a common compartment of extracellular fluid (ECF) [36,37]. Utilizing ^42^K+ intravenous (IV) injections, researchers noted a wide spectrum of tracer exchange rates between tissues, with kidneys being the fastest (equilibrium with plasma at 2 min) and muscle and brain being the slowest (≥600 min) [36]. Based on this model, total potassium mass of the four primary tissue compartments should be equivalent to total body potassium. However, findings revealed that this was not the case, the total sum only accounting for 73% of potassium mass. Investigators concluded that exchange rates/pools may be heterogeneous across both organs and organ groups, making the idea of grouping tissue compartments even more complex, and the internal movement of potassium more nuanced. Later, Leggett and Williams proposed a more anatomically specific model based on the quantitative movement of potassium through mathematically derived compartments within a physiologically relevant framework [38]. Their model, similar to previous depictions, identifies plasma/ECF as the primary feeding compartment, with equilibrium distribution of potassium, regional blood flow rates, and potassium tissue extraction fractions, all influencing potassium exchange. The model also describes potassium exchange from plasma/ECF to tissues as a relatively rapid and uniform process; skeletal muscle being the only exception, with slower exchange due to its role as the main site of potassium storage [38]. This concept is confirmed by earlier studies looking at exchange rates of total body potassium using measures of whole body counting of radioactivity, IV administration of ^42^K, and ^40^K/^42^K ratios [39,40]. These early works revealed that, after absorption, most body potassium exchanges rapidly with a half-life of less than 7 h, while a small portion thought to be contained primarily in skeletal muscle exchanges more slowly (~70 h) [40,41]. A better understanding of kinetic modeling and potassium movement throughout the body may help to reveal how specific tissues influence potassium bioavailability, and further the understanding of its role in health.

In a recent study conducted by Macdonald and colleagues, researchers aimed to assess and compare the bioavailability of potassium from potato sources (non-fried white potatoes, French fries) and a potassium supplement (potassium gluconate) [6] Thirty-five healthy men and women (29.7 ± 11.2 years, 24.3 ± 4.4 kg/m^2^) were randomized to nine, five-day interventions of additional K+ equaling: 0 mEq (control at phase 1 and repeated at phase 5), 20 mEq (1500 mg), 40 mEq (3000 mg), 60 mEq (4500 mg) K+/day consumed as K+ gluconate or potato, and 40 mEq K+/day from French fries. Bioavailability of potassium was determined from serum AUC (serial blood draws) and 24 h urinary excretion assessed after a test meal of varying potassium dose given on the 4th day. Investigators found increases in serum potassium AUC with increasing dose regardless of source, while potassium 24 h. urine concentration also increased with dose but was greater with potato compared to supplement. Blood pressure (BP) was also assessed throughout the study but resulted in no significant findings. These outcomes reveal the need for a full potassium balance study, looking at intakes from a variety of dietary sources and complete losses (urine and feces), to fully understand potassium bioavailability differences between dietary potassium and supplements and their subsequent health effects.

## 5. Potassium and Hypertension

Hypertension (HTN) is the leading cause of cardiovascular disease (CVD) and a major contributing risk factor for the development of stroke, coronary heart disease (CHD), myocardial infarction, heart failure, and end-stage renal disease, amounting to a US public health financial burden of $50.6 billion [42]. Nearly one in three American adults (~72 million) are estimated to have HTN, while nearly 70 million are at risk for developing pre-hypertension (BP between 120/80 mmHg and 140/90 mmHg). Approximately 90% of US adults older than 50 are at risk for the development HTN, with systolic rises being the most prevalent [43]. Hypertension is a leading cause of morbidity and mortality worldwide and second only to smoking as a preventable cause of death in the United States [44].

### 5.1. Epidemiological Data

Numerous epidemiological studies show diet as a key component in blood pressure (BP) control, with some studies showing lower BP in populations consuming higher amounts of fruits and vegetables [45,46,47]. Dietary patterns known to lower BP include reduced sodium intake, increased potassium and magnesium intake, increases in fruit and vegetable consumption, as well as other foods rich in antioxidants [48,49]. A population study conducted by Khaw et al. (1982) in St. Lucia, West Indies suggested an increase in potassium by 20–30 mmol/day (~700–1200 mg/day), which resulted in a 2 to 3 mmHg reduction in systolic blood pressure (SBP) [50]. In adults, a 2-mmHg reduction in BP can reduce CHD and stroke mortality rates by 4 and 6%, respectively [51]. In addition, as revealed through the INTERSALT study, the Yanomami Indians of Brazil consumed a low sodium, high potassium (mostly vegetarian) diet and had low average BP, lack of BP rise with age, and no HTN [52]. Overall, the INTERSALT study provided evidence of potassium intake as an important factor effecting population BP, independent of sodium, among diverse population groups [51]. The American Heart Association has estimated that increasing potassium intake may decrease HTN incidence in Americans by 17% and lengthen life span by 5.1 years [42]. Attaining adequate potassium intake may be the most influential dietary component in lowering BP, with a diet containing >3500 mg/day recommended for primary prevention of HTN [53].

### 5.2. Observational Research

Observational studies have evaluated the effects of potassium from foods, while clinical intervention trials have primary used potassium supplements. Several meta-analyses show a significant reduction in BP with increasing potassium supplementation [54,55,56,57]. In an early meta-analysis, Cappuccio and MacGregor reviewed 19 clinical trials looking at the effect of potassium supplementation on BP in primarily hypertensive individuals (412 of 586 participants). With the average amount of potassium given at 86 mmol/day (~3300 mg/day; as primarily KCl) for an average duration of 39 days, researchers found that potassium supplementation significantly reduced SBP by 5.9 mmHg and diastolic blood pressure (DBP) by 3.4 mmHg. Greater reductions were found in individuals who were on supplementation for longer periods of time [56]. Another regression-analysis looked at the effect of potassium supplementation in both normotensive and hypertensive individuals. Researchers found an average potassium dose of 60–120 mmol/day (2500–5000 mg/day) reduced SBP and DBP by 4.4 and 2.5 mmHg, respectively, in hypertensive patients, and by 1.8 and 1.0 mmHg, respectively, in normotensive individuals [55]. As is evident, the effect of potassium supplementation on BP reduction is generally positive, but not consistent. According to a more recent meta-analysis conducted by Dickinson et al., potassium supplementation did not significantly reduce BP in those with hypertension, although this analysis was only based on five trials, and findings, while not statistically significant, did reveal reductions in both SBP and DBP [54,58]. In general, these outcomes show that the BP lowering effects of potassium supplementation are greater in those with HTN and more pronounced in blacks compared to whites. Other noted factors that may influence the effects of potassium supplementation on BP include pre-treatment BP, age, gender, intake of sodium and other ions (magnesium, calcium), weight, physical activity level, and concomitant medications. In addition, these analyses suggest a relationship between an optimal potassium dose range (between 1900 and 3700 mg/day) and BP lowering of approximately 2–6 mmHg in SBP and 2–4 mmHg in DBP [59]. However, this was not confirmed in the previously described dose-response bioavailability study, although all participants were normotensive and duration was short [6].

### 5.3. Clinical Findings

Overall findings from clinical trials on the effect of increased potassium intake have been conflicting [43,48,60,61]. Evidence from dietary interventions is extremely limited, with the majority of findings being extrapolated from The Dietary Approaches to Stop Hypertension (DASH) study [48]. The DASH intervention revealed that a diet rich in fruit and vegetables, fiber, and low fat dairy products, with reductions in saturated and total fat and sodium could positively influence BP compared to the average American diet [62]. Although the DASH diet does lead to a dramatic increase in potassium consumption (+1447 to 2776 mg/day) and reduction in BP, due to its other dietary modifications, these beneficial effects cannot be attributed to potassium alone [49]. In an earlier study conducted by Chalmers et al., researchers assessed the effects of both the reduction of dietary sodium and increase of dietary potassium on BP [63]. Two-hundred-and-twelve subjects (age 52.3 ± 0.8 years; 181 males and 31 females) with a DBP between 90 and 100 mmHg were recruited and placed in one of the 4 following diet groups: a normal diet group (control), a high potassium diet (>100 mmol·K/day; >3900 mg/day), a reduced sodium diet (50–75 mmol·Na+/day; 1150–1725 mg/day), or a high potassium/low sodium diet. Subjects completed the diet phase for 12 weeks in which they were regularly counseled on how to adequately modify their food choices based on their group (e.g., avoiding salt and high sodium foods, increasing fruit and vegetable intake). Investigators found significant reductions in both SBP and DBP in each dietary intervention group compared to controls, but no significant differences between groups, with reductions in the high potassium group being 7.7 ± 1.1 and 4.7 ± 0.7 mmHg for SBP and DBP, respectively. Although high potassium intake did appear to reduce BP the lack of differences between groups points to the possibility of an overall diet effect. In addition, for both the high potassium and low sodium groups, there was a significant reduction in weight during the study, which may have further confounded the results. In a more recent study, Berry and colleagues assessed the effects of increased potassium intake from both dietary sources and supplements on BP in untreated pre-hypertensive individuals (DBP between 80 and 100 mmHg) [64]. In a cross-over design, subjects (*n* = 48, 22–65 years) completed four, six-week dietary interventions including a control diet, an additional 20 or 40 mmol·K/day (780 or 1560 mg/day) from fruit and vegetables, and 40 mmol potassium citrate/day capsules. Each treatment period was followed by a washout period of no less than five weeks. Similar to the Chamlers study, nutrition coaching was used to regulate participant food choice during each dietary intervention, primarily focused on increasing fruit and vegetable intake (which excluded potassium rich potatoes). Findings revealed no significant changes in ambulatory BP between the control group and any of the dietary or supplement interventions. The lack of control used to conduct these potassium dietary interventions is the primary limiting factor in their ability to adequately assess the true effect of increased dietary potassium intake on BP outcomes. A complete balance study with a controlled diet is necessary to accurately assess potassium retention, and its acute and prolonged effects on BP and other health outcomes. A summary of the clinical findings on the effects of increased potassium intake on BP measures (SBP and DBP) can be found in Table S1. 

### 5.4. Mechanisms in Which Potassium May Improve Vascular Outcomes

The antihypertensive effect of increased potassium intake is related to numerous mechanisms. Acutely, increased plasma potassium is associated with endothelium dependent vasodilation via stimulation of Na+-K+ ATPase pumps and the opening of potassium channels in vascular smooth muscle cells and adrenergic nerve receptors [65]. Long-term potassium dosing induces increases in the number of Na+-K+ ATPase pumps in basolateral cell membranes and increases in the transepithelial voltage. Increased pumping can result from either increased Na+-K+ ATPase turnover (acute K+ loading) or an increase in the number of pumps (long-term K+ loading), or both [65]. In addition to enhanced vasodilation, other possible mechanisms in which potassium is proposed to lower BP and improve vascular outcomes include increases in sodium excretion, modulation of baroreceptor sensitivity, reduced sensitivity to catecholamine related vasoconstriction, improved insulin sensitivity, and decreases in oxidative stress and inflammation [59].

## 6. Potassium and Glucose Intolerance, Insulin Sensitivity, and Diabetes

Glucose intolerance can often be a result of severe hypokalemia due to a deficit in potassium balance that may occur in primary or secondary aldosteronism or prolonged treatment with diuretics [5]. The use of thiazide diuretics are widely considered the preferred initial pharmacological treatment for hypertension [66]. The tendency of thiazide diuretics to negatively influence glucose tolerance and increase the incidence of new onset diabetes has been known [67]. In a recent quantitative review, researchers analyzed 59 clinical trials in which the relationship between the use of thiazide diuretics, hypokalemia, and glucose intolerance was strong [66]. Thiazide diuretics have a common side effect of lowering serum potassium and evidence shows that diuretic-induced hypokalemia may lead to impaired glucose tolerance via reduction in insulin secretion in response to glucose loads [68]. In healthy individuals, there is also evidence to support the role of potassium in glucose control. Studies involving potassium depletion (e.g., low potassium diets) and utilizing a hyperglycemic clamp technique show that low levels of potassium can lead to glucose intolerance via impaired insulin secretion [69,70]. Potassium’s role in the control of blood glucose is grounded in its function at a cellular level where potassium-induced cell depolarization results in insulin secretion from pancreatic β-cells [71]. In addition, when patients with thiazide-induced hypokalemia are given potassium supplements, the defects in insulin release in response to glucose loads are corrected, thus indicating that hypokalemia may be a significant contributing factor to the glucose abnormality [72]. 

The relationship between potassium intake and diabetes was further examined in a prospective cohort study conducted by Colditz and colleagues (1992) looking at women (*n* = 84,360; 34–59 years) from the Nurse’s Health Study. After a six-year follow-up, investigators found that high potassium intake may be associated with a decreased risk for developing type 2 diabetes mellitus (T2DM) in women with a BMI of 29 or less [73]. When compared with women in the lowest quintile, women in the highest quintile for potassium intake had a relative risk of 0.62 (*p* trend = 0.008) for T2DM. More recently, Chatterjee et al. assessed the association between potassium intake and T2DM using data from the Coronary Artery Risk Development in Young Adults (CARDIA) study [74]. Researchers examined the relationship between urinary potassium and diabetes risk for 1066 participants. Use of multivariate models adjusted for potential confounders including BMI, fruit and vegetable intake, and other dietary factors revealed that those in the lowest quintile of potassium intake were more than twice as likely to develop diabetes compared to those in the highest quintile (HR 2.45; 95% CI 1.08, 5.59; *p* for trend 0.04) [74]. Investigators also found that those in the lowest quintile of potassium intake were significantly more likely to develop diabetes than those in the highest quintile of potassium intake (*p* = 0.008). Of the 4754 participants, 373 (7.8%) developed diabetes during the follow up period of 20 years, and, overall, the mean potassium intake of those who developed diabetes was significantly lower than those who did not (3393 mg/day vs. 3684 mg/day; *p* = 0.002) [74]. This same research group examined data from 12,209 individuals participating in the Atherosclerosis Risk in Communities (ARIC) cohort and found serum potassium to be independently associated with diabetes risk. Using multivariate cross-sectional analyses, a significant inverse relationship between serum potassium and fasting insulin levels was identified (*p* < 0.01) [75]. Dietary potassium intake was significantly associated with diabetes risk in unadjusted models, with adults having serum potassium levels lower than 4.0 mEq/L at highest risk for DM incidence. This relationship continued to hold true after covariate adjustment (e.g., age, sex, race, BMI, serum magnesium, serum calcium, physical activity, hypertension, etc.) in multivariate models, with lower potassium levels associated with higher BMI, larger waist circumference, lower serum magnesium levels, and higher fasting insulin levels as well [75].

The relationship between potassium and T2DM also extends to the kalemic effects of insulin. Higher plasma insulin levels are associated with increased potassium absorption into cells [76], and without a threshold as seen in glycemic response, these kalemic effects continue to increase as insulin levels rise [77]. DeFronzo et al. [76] examined this relationship using the insulin clamp technique and graded doses of insulin. Researchers found that insulin caused a dose-dependent decline in plasma potassium concentration, and that this relationship was independent of glucose uptake [76]. This effect is also seen through the use of IV insulin as a treatment for hyperkalemia, forcing potassium into cells and thus reducing its concentration in the blood [77]. This is likely to be mediated by an increased sensitivity to intracellular sodium, activation of Na+-K+ ATPase, and inhibition of potassium efflux [76].

## 7. Conclusions

Increasing dietary potassium has a potential benefit for lowering the risk of hypertension, the major risk factor for the development of stroke, coronary heart disease, heart failure, and end-stage renal disease. In addition, adequate potassium intake may be extremely influential in glucose control and limiting the risk of diabetes, especially in those on thiazide diuretic treatment, and those already at higher risk from the development of additional co-morbidities. Increasing evidence suggests these changes in dietary habits would also have potential health benefits for the skeleton and kidneys. Potassium supplementation and potassium fortification are less attractive as approaches to increasing potassium intake than for other vitamins and minerals. The quantity of potassium needed to fill the gap requires large amounts of supplements or fortification, and the compounds are bitter, thus having low palatability. Furthermore, some of these compounds can pose health risks (e.g., potassium bromate has been used as food additive and is still used in barely processing) and are banned in some countries such as the United Kingdom [78].

We need to understand more about bioavailability of potassium from foods. Only the potato has been studied for potassium bioavailability, and this food is constituted mostly of easily digested starch. Are there as of yet unidentified inhibitors to potassium absorption or food matrix effects? Do some anions that accompany potassium in foods have differential functional advantages? Organic salts of potassium appear to have more benefit to bone, perhaps through effects on acid-base balance. The form seems less important for controlling for blood pressure. Potassium is likely a nutrient with forthcoming research because it is an identified shortfall nutrient. It is a well-established modifiable factor for hypertension, the largest risk of some of our most common chronic diseases, and a better understanding of its bioavailability in the diet will help determine how it can be used further to improve overall human health.

## Supplementary Materials

The following are available online at http://www.mdpi.com/2072-6643/8/7/444/s1, Table S1: Published studies that showed an effect of additional potassium (K+) intake (supplement or dietary) on blood pressure (BP) outcomes.
