# Supplementary material for: Potassium Intake, Bioavailability, Hypertension, and Glucose Control
Source: Nutrients. 2016 Jul 22;8(7):444. doi: 10.3390/nu8070444 (PMC4963920; doi:10.3390/nu8070444)
Supplement: Supplementary file 1 [file nutrients-08-00444-s001.docx]

Supplementary Materials: Potassium Intake, Bioavailability, Hypertension, and Glucose Control

Michael S. Stone, Lisa Martyn and Connie M. Weaver

**Table S1.** Published studies that showed an effect of additional potassium (K+) intake (supplement or dietary) on blood pressure (BP) outcomes.

| **First Author, Date** | **Population** | **Design** | **Duration** | **K+ Form** | **K+ Dose** | **BP Outcome** |
| --- | --- | --- | --- | --- | --- | --- |
| Cappuccio & MacGregor, 1991 [1] | Participants: 586  (412 hypertensives),  men (76%) and women,  18–66 years (mean: 39.6). | Meta-Analysis of  19 clinical trials. | 5 days–16 weeks. | K+ supplement. | 48–140 mmol/day  (1872–5460 mg/day). | Reduction in SBP  (−5.9 mmHg) and DBP  (−3.4 mmHg). |
| Whelton et al., 1997 [2] | Participants: 2609  (1560 hypertensives), men and women, 18–79 years. | Meta-Analysis of  33 clinical trials. | 4 days–3 years;  mean 5 weeks. | K+ supplement. | 60–100 mmol/day  (2340–3900 mg/day);  median 75 mmol/day (2925 mg/day) | Reduction in SBP  (−3.11 mmHg) and DBP  (−1.97 mmHg). |
| Geleijnse et al., 2003 [3] | Men (60%) and women,  45 ± 12 years. | Meta-Analysis of  30 clinical trials. | 2–114 weeks;  mean 6 weeks. | K+ supplement. | Not given | Reduction in SBP  (−2.42 mmHg) and DBP  (−1.57 mmHg). |
| Dickinson et al., 2006 [4] | Participants: 425, men (75%) and women, 36–52 years,  all hypertensives. | Meta-Analysis of  5 clinical trials. | 8–16 weeks;  mean 12 weeks. | K+ supplement. | 48–120 mmol/day  (1872–4680 mg/day). | No significant reduction in  SBP or DBP. |
| Chalamers et al., 1986 [5] | Participants: 212, men (181)  and women (31),  mean age 52.3 ± 0.8 years),  all hypertensives  (DBP: between 90 and  100 mmHg). | Clinical trial with 4 arm parallel design: normal (control) diet, high K+ diet, low Na+ diet, and high K+/low Na+ diet.  (2 × 2 factorial). | 8 week run-in (control diet) followed by  12 weeks of intervention. | K+ from increased intake of K+ rich dietary sources (free living diet, subjects counseled on what foods to eat depending on group). | K+ content of intervention diets: high potassium and  high potassium/low sodium- >100 mmol/day (3900 mg/day), control/low sodium-  not reported. | High K+ diet: SBP and DBP: −7.7 and −4.7, respectively.  Low Na+ diet: −8.9 and −5.8.  High K+/low Na+  diet: −7.9 and −4.2.  All compared to control. |
| Appel et al., 1997  (DASH Trial) [6] | Participants: 459, men and women (~50% each),  >22 years  (mean: ~45 ± 10),  SBP: <160 mmHg,  DBP: 80–95 mmHg. | Clinical trial with 3 arm parallel design: control diet, high fruit and vegetable diet, high fruit and vegetable diet + reduced fat  (combination diet). | 3 weeks control diet run-in, followed by  8 weeks of intervention. | K+ from increased intake of from fruits and vegetables  (from controlled  research diets). | K+ content of each diet: Control- 1752 mg/day, High fruit and vegetable- 4101 mg/day, Combination diet- 4415 mg/day. | Combination diet: SBP and DBP:  −5.5 and −3.0 mmHg, respectively. High fruit and vegetable diet: SBP and DBP: −2.8 and  −1.1 mmHg, respectively.  Both compared to control. |

**Table S1.** *Cont.*

| **First Author, Date** | **Population** | **Design** | **Duration** | **K+ Form** | **K+ Dose** | **BP Outcome** |
| --- | --- | --- | --- | --- | --- | --- |
| Berry et al., 2010 [7] | Participants: 48, men (23)  women (25), 22–65 years  (mean age: ~45 ± 10 years).  All hypertensives (DBP: between 80 and 100 mmHg). | Clinical trial cross-over design with 4 interventions: control diet, control diet + K+ supplement, additional intake (20 mmol/day) from fruits and vegetables, additional intake  (40 mmol/day) from fruits  and vegetables. | 3 week run-in (control diet), four 6 week diet interventions with at least 5 weeks washout in between. | K+ supplement, K+ from increased intake of fruits and vegetables (free living diet, subjects counseled on what foods to eat depending on group). | K+ content of intervention diets: control diet: 15 mmol/day, control diet + potassium supplement:  40 mmol/day; 1560 mg/day, control diet + additional intake from fruits and vegetables:  20 mmol/day; 780 mg/day and  40 mmol/day; 1560 mg/day. | No significant change in SBP or DBP within interventions  (pre, post) or  compared to control. |
| Macdonald-Clarke et al., 2016 (in press) [8] | Participants: 35, men and women, 20–60 years  (mean age: 29.7 ± 11.2), normotensives. | Single-blind, cross-over, randomized control trial with 9 interventions of additional potassium:  0 mmol/day, 20 mmol/day,  40 mmol/day from supplement or potato, and 40 mmol/day from French fries. | 5 day periods of additional potassium, separated by at least 7 days of washout. | K+ supplement, K+ from increased intake of potatoes and French Fries (from controlled research diets). | K+ content (additional) of intervention diets: 0 mmol/day (control = 60 mmol/day total; phases 1 and 5), 20, 40, 60 mmol/day (780, 1560, 2340 mg/day) from supplement or potato, 40 mmol/day (1560 mg/day) from French Fries. | No significant change in SBP or  DBP within interventions or compared to control. |

BP, blood pressure; DBP, diastolic blood pressure; K+, potassium; Na+, sodium; SBP, systolic blood pressure; All BP outcomes statistically significant unless otherwise noted.

**References**

1. Cappuccio, F.P.; MacGregor, G.A. Does potassium supplementation lower blood pressure? A meta-analysis of published trials. *J. Hypertens.* **1991**, *9*, 465–473.
2. Whelton, P.K.; He, J.; Cutler, J.A.; Brancati, F.L.; Appel, L.J.; Follmann, D.; Klag, M.J. Effects of oral potassium on blood pressure. Meta-analysis of randomized controlled clinical trials. *JAMA* **1997**, *277*, 1624–1632.
3. Geleijnse, J.M.; Kok, F.J.; Grobbee, D.E. Blood pressure response to changes in sodium and potassium intake: A metaregression analysis of randomised trials. *J. Hum. Hypertens.* **2003**, *17*, 471–480.
4. Dickinson, H.O.; Nicolson, D.J.; Campbell, F.; Beyer, F.R.; Mason, J. Potassium supplementation for the management of primary hypertension in adults. *Cochrane Database Syst. Rev.* **2006**, CD004641, doi:10.1002/14651858.CD004641.pub2.
5. Chalmers, J.; Morgan, T.; Doyle, A.; Dickson, B.; Hopper, J.; Mathews, J.; Matthews, G.; Moulds, R.; Myers, J.; Nowson, C.; et al. Australian national health and medical research council dietary salt study in mild hypertension. *J. Hypertens. Suppl.* **1986**, *4*, S629–S637.
6. Appel, L.J.; Moore, T.J.; Obarzanek, E.; Vollmer, W.M.; Svetkey, L.P.; Sacks, F.M.; Bray, G.A.; Vogt, T.M.; Cutler, J.A.; Windhauser, M.M.; et al. A clinical trial of the effects of dietary patterns on blood pressure. Dash collaborative research group. *N. Engl. J. Med.* **1997**, *336*, 1117–1124.
7. Berry, S.E.; Mulla, U.Z.; Chowienczyk, P.J.; Sanders, T.A. Increased potassium intake from fruit and vegetables or supplements does not lower blood pressure or improve vascular function in uk men and women with early hypertension: A randomised controlled trial. *Br. J. Nutr.* **2010**, *104*, 1839–1847.
8. Macdonald-Clarke, C.J.; Martin, B.R.; McCabe, L.D.; McCabe, G.P.; Lachcik, P.J.; Wastney, M.; Weaver, C.M. Bioavailability of potassium from potatoes and potassium gluconate: A randomized dose response trial. *Am. J. Clin. Nutr.* **2016**, in press.

© 2016 by the authors; licensee MDPI, Basel, Switzerland. This article is an open access article distributed under the terms and conditions of the Creative Commons by Attribution (CC-BY) license (http://creativecommons.org/licenses/by/4.0/).
